# The application of high-density genetic maps of rye for the detection of QTLs controlling morphological traits

**DOI:** 10.1007/s13353-013-0186-5

**Published:** 2013-12-03

**Authors:** Beata Myśków, Monika Hanek, Aneta Banek-Tabor, Robert Maciorowski, Stefan Stojałowski

**Affiliations:** 1 Department of Plant Genetics, Breeding and Biotechnology, West Pomeranian University of Technology, Słowackiego 17, 71-434 Szczecin, Poland; 2 Research Institute of Horticulture, Konstytucji 3 Maja 1/3, 96-100 Skierniewice, Poland

**Keywords:** Genetic maps, Morphology, Yield structure, QTL, Rye

## Abstract

The development of genetic maps is, nowadays, one of the most intensive research activities of plant geneticists. One of the major goals of genome mapping is the localisation of quantitative trait loci (QTLs). This study was aimed at the identification of QTLs controlling morphological traits of rye and comparison of their localisation on genetic maps constructed with the use of genetically different germplasms. For QTL analyses, two high-density consensus maps of two populations (RIL-S and RIL-M) of recombinant inbred lines (RIL) were applied. Plant height (Ph), length of spikes (Sl) and the number of spikelets per spike (Sps) were studied in both populations. Additionally, the number of kernels per spike under isolation (Kps), the weight of kernels per spike (Kw) and thousand kernel weight (Tkw) were assessed in the RIL-M population. Except for Tkw, the majority of the traits were correlated to each other. The non-parametric Kruskal–Wallis (K-W) test and composite interval mapping (CIM) revealed 18/48 and 24/18 regions of rye chromosomes engaged in the determination of Ph, Sl and Sps in the RIL-S and RIL-M populations, respectively. An additional 18/15 QTLs controlling Kps, Kw and Tkw were detected on a map of the RIL-M population. A numerous group of QTLs detected via CIM remained in agreement with the genomic regions found when the K-W test was applied. Frequently, the intervals indicated by CIM were narrower.

**Electronic supplementary material:**

The online version of this article (doi:10.1007/s13353-013-0186-5) contains supplementary material, which is available to authorized users.

## Introduction

The development of genetic maps of cultivated species has been one of the most intensive research activities for plant geneticists over the last two decades. One of the major goals of genome mapping projects is the localisation and characterisation of quantitative trait loci (QTLs) controlling important agronomical traits. The identification of QTLs opens a possibility for marker-assisted selection (MAS) and facilitates the study of changes in the genomes of plants experienced during domestication and breeding (Varshney et al. 2004). In rye, the first genetic map of all seven chromosomes was released 20 years ago by Devos et al. (1993a). Since that time, several genetic maps of the rye genome have been published (Philipp et al. 1994; Senft and Wricke 1996; Korzun et al. 2001; Ma et al. 2001; Hackauf and Wehling 2003; Khlestkina et al. 2004; Milczarski et al. 2007), but until 2009, all of them were of low density. Recently, two types of research activities have had a strong impact on the mapping progress in rye. The first was the application of new software allowing joint exploration of data from different mapping populations and the construction of integrated/consensus maps of chromosomes (Gustafson et al. 2009; Stojałowski et al. 2009). The second was the development of a new molecular marker system for rye based on a very efficient method of polymorphism detection, i.e. Diversity Arrays Technology (Bolibok-Brągoszewska et al. 2009). Finally, as a result of application of the novel marker systems and mapping software, a consensus genetic map of rye, which considers segregation data from five mapping populations and includes over 4,000 loci, was published (Milczarski et al. 2011).

Despite the significant progress made in the construction of genetic maps of the rye genome, there are still limited data available regarding the inheritance of important agronomical traits. The first report regarding the identification of QTLs for morphological traits in rye was published by Börner et al. (2000), followed by that of Milczarski and Masojć (2003). In both studies, loci determining morphological and yield-related traits were identified with the use of low-density maps on all rye chromosomes; nevertheless, the strongest impact on several traits was noted for genes localised on the long arm of chromosome 5R. Recently, a set of 440 test-crosses (Miedaner et al. 2012) was applied for the identification of genome regions responsible for several agronomic and quality traits in rye. Nonetheless, knowledge about the genetic determination of quantitative traits in rye is still very limited and insufficient for breeding purposes.

In this study, high-density genetic maps constructed for two populations of recombinant inbred lines (RIL) were used for the identification of QTLs engaged in the expression of morphological traits of agronomical importance. For QTL analyses, consensus maps previously constructed by Milczarski et al. (2011) were applied. Two of the five mapping RIL populations used for the construction of consensus maps were chosen for this research. They represented the highest and lowest genetic variation. The present study was aimed at the identification of QTLs controlling morphological and yield-related traits in rye and a comparison of their localisation on genetic maps constructed with the use of genetically different germplasms.

## Materials and methods

### Plant material and genetic maps

The plant material used in this study represents two RIL populations. The first population, RIL-S, was obtained from a cross between inbred lines 541 and 2020LM, while the second, RIL-M, originates from a cross between lines S120 and S76. The pedigree of line 541 is complex and one of its ancestral forms is a wild perennial rye *Secale montanum* (Łapiński and Stojałowski 1996). The three remaining inbred lines were developed within breeding programmes conducted at the Institute of Plant Breeding and Acclimatization (Radzików, Poland) and DANKO Plant Breeding Ltd. (Choryń, Poland), and kindly provided for our study by L. Madej and W. Brukwiński. Parental lines used for the development of the RIL-S population are unrelated, whereas the lines used for the development of the RIL-M population are partially related, yet genetically different (Myśków et al. 2001). Analyses applying molecular markers (Milczarski et al. 2011) confirmed the pedigree data: the genetic similarity of lines 541 and 2020LM was estimated at 0.46, and lines S120 and S76 at 0.35.

Consensus genetic maps for each mapping population (based on an analysis of the data from all populations) were created using the Multipoint Consensus 2.2 software package (Korol et al. 2009). Detailed information on the RIL-S and RIL-M mapping populations and algorithms used for releasing the maps is found in Milczarski et al. (2011).

### Phenotype analyses

All experimental trials with RILs and parental lines were conducted on the experimental fields of the West Pomeranian University of Technology in Szczecin. The RIL-M population (generations F_8_–F_10_) consisted of 143 lines and was analysed in three vegetation seasons (years 2008–2010). The 92 lines of the RIL-S population (F_6_–F_9_) were assessed over four seasons (2008–2011). Due to a high inbreeding depression of numerous lines, individuals representing each line were first germinated in a glasshouse and then vital seedlings were planted manually in the field, with each line planted in two adjacent rows. Finally, eight individuals were grown in each row (the length of rows was 1 m and the distance between rows was 18 cm). The order of lines grown in the field was random and different in each year of the study. Between five and eight randomly chosen individual plants were analysed and considered to be replications in the statistical analysis. The following traits were studied: plant height (Ph), length of spikes (Sl) and the number of spikelets per spike (Sps). Additionally, the number of kernels per spike under isolation (Kps), the weight of kernels per spike (Kw) and thousand kernel weight (Tkw) were determined in the RIL-M population. In the RIL-S population, these traits were omitted because over 25 % of lines revealed very low pollen shedding, leading to a high sensitivity of seed setting to environmental conditions (rainy/sunny weather) occurring at the flowering time of a given spike. As a consequence, very high variation was observed between seed settings within isolated spikes, leading to a low precision of phenotyping.

### Statistical analysis

Statistical analyses (means, standard deviations, correlation coefficients) were calculated using STATISTICA v.9.0 software (http://www.statsoft.com). The significance of differences between parental lines was established by employing the Cochran and Cox test. Variance components were estimated using the restricted maximum likelihood method (REML) and broad-sense heritabilities (h^2^_BS_) were approximated using the following formula (Holland et al. 2003):

$$ {{\mathrm{h}}^2}_{\mathrm{BS}}={\sigma^2}_G/\left({\sigma^2}_G+{\sigma^2}_Y+{\sigma^2}_{GY}+{\sigma^2}_E\right) $$

where *σ* ^2^ are estimators of variance components associated with genotypic (*G*), seasonal (*Y*), genotype–year interaction (*GY*) effects and experimental error (*E*).

Relationships between the segregation of molecular markers and studied traits were analysed with the Kruskal–Wallis (K-W) test using the MapQTL 5.0 package (Van Ooijen 2004). Genomic regions were considered to contain QTLs if, in at least two vegetation seasons, the significance of molecular markers (at *P* < 0.01) was recorded.

Verification of the QTL mapping was performed using the composite interval mapping (CIM) method with Windows QTL Cartographer 2.51 software (http://statgen.ncsu.edu/qtlcart/WQTLCart.htm; Wang et al. 2007). The step size chosen for all QTLs was 2 cM. Thresholds for declaring the presence of QTLs were estimated from 1,000 permutations of the data (Doerge and Churchill 1996) for each trait and year of study; therefore, the significant level of LOD varied from 1.8 to 2.7.

## Results

Parental lines of the RIL-S population (inbred lines 541 and 2020LM) differed significantly for all of the studied traits (Table 1). In turn, phenotypic differences between the inbred lines S120 and S76, which were used for the development of the RIL-M population, were not large and, in the majority of cases, not significant. Nevertheless, phenotypic variation was observed in both of the mapping populations. The range of this variation was dependent on the year of study, but, generally, in the RIL-S population, it did not significantly exceed the mean values found within its parental lines. Interestingly, in the RIL-M population, being representative for a narrow genetic variation among its parents, the phenotypic variation was comparable to that observed in the RIL-S population. For all of the studied traits, the range of variation within RIL-M was significantly wider than the differences between its parental lines S120 and S76, suggesting the presence of transgression.

**Table 1:** Phenotypic expression of morphological traits within the studied populations, characterization of variance components and estimation of heritability (h^2^_BS_)

Population		RIL-S (541 × 2020LM)			RIL-M (S120 × S76)					
Trait		Ph	Sl	Sps	Ph	Sl	Sps	Kps	Kw	Tkw
Parental lines^a^ Mean (SD)	Maternal	124.1 (9.7)	10.7 (0.6)	34.4 (3.3)	106.8 (9.7)	7.8 (1.1)	26.4 (4.3)	21.8 (11.1)	0.41 (0.31)	17.2 (6.5)
	Paternal	68.1 (12.9)	5.4 (1.6)	22.7 (6.1)	89.6 (10.6)	7.2 (0.9)	26.5 (2.9)	29.1 (10.7)	0.71 (0.35)	23.8 (5.3)
Mapping population Mean (SD) *Variation range*	2008	112.0 (14.0) *81.2–141.6*	8.6 (1.3) *5.5–12.0*	31.5 (3.8) *22.4–38.0*	99.0 (11.5) *67.0–125.0*	8.3 (1.0) *5.9–11.5*	30.3 (3.2) *18.0–38.8*	28.0 (10.4) *3.2–51.8*	0.8 (0.3) *0.08–1.57*	27.2 (5.2) *15.5–39.3*
	2009	91.7 (18.8) *41.0–134.8*	6.9 (1.4) *3.5–10.4*	24.7 (3.7) *14.0–35.2*	95.4 (10.4) *58.3–121.5*	6.9 (0.8) *4.4–9.3*	24.7 (2.4) *18.7–31.0*	27.4 (7.1) *8.7–43.8*	0.7 (0.2) *0.15–1.21*	23.9 (4.9) *11.4–35.9*
	2010	88.4 (16.2) *54.0–124.2*	7.4 (1.4) *3.7–11.6*	25.2 (4.1) *10.7–34.3*	98.9 (21.6) *42.5–123.3*	7.6 (1.2) *4.3–11.2*	26.6 (3.0) *17.3–33.1*	18.3 (9.2) *1.4–43.4*	0.4 (0.2) *0.03–1.07*	20.0 (5.3) *7.5–49.8*
	2011	104.9 (13.1) *80.0–141.3*	8.2 (1.1) *5.8–10.5*	28.0 (3.1) *21.1–35.0*						
Correlation coefficient between years of study (min–max)		0.65–0.86	0.63–0.76	0.42–0.66	0.74–0.81	0.59–0.66	0.55–0.60	0.52–0.60	0.53–0.62	0.45–0.54
Components of variance^b^ (%)	Years (Y)	29.35*	17.52*	28.77*	1.49	22.45*	33.43*	17.76*	27.68*	29.31*
	Genotypes (G)	45.14*	39.12*	25.58*	66.29*	36.24*	25.22*	35.86*	31.38*	28.75*
	Y × G	12.50*	12.72*	14.10*	9.40*	13.39*	10.95*	15.67*	13.25*	15.09*
h^2^_BS_		0.45	0.39	0.26	0.66	0.36	0.25	0.36	0.31	0.29

*Ph* plant height (cm), *Sl* spike length (cm), *Sps* number of spikelets per spike, *Kps* number of kernels per spike, *Kw* kernel weight per spike (g), *Tkw* thousand kernel weight (g)

^a^Significant differences between parental lines (based on the Cochran and Cox test) are exhibited by underlined font type

^b^Components of variance estimated by the restricted maximum likelihood (REML) method (Holland et al. 2003). Significance of Y, G and Y × G is indicated by asterisks

Environmental conditions had a substantial influence on the expression of all analysed traits. Broad-sense heritabilities for the studied morphological features varied between 0.25 and 0.66. The highest heritabilities were recorded for Ph in both mapping populations, while Sps revealed the lowest heritability. Genotypic components of variance were statistically significant for all of the analysed traits.

The general means of data collected in different years were significantly correlated for all analysed traits, confirming the importance of genetic components of variance (Table 1). The majority of the studied traits were correlated to each other (Table 2). The strongest correlations were observed between Sl and Sps, as well as between Kps and Kw. Markedly weaker correlations were noticed between Tkw and the majority of the other traits (the only exception was Kw).

**Table 2:** Correlation coefficients between studied traits assessed in the years of study (range: min–max). Results from the RIL-S population are shown above the diagonal, and those from RIL-M below the diagonal

	Ph	Sl	Sps	Kps	Kw
Ph	×	0.40^3^–0.64^3^	0.32^1^–0.60^3^		
Sl	0.26^1^–0.43^3^	×	0.77^3^–0.88^3^		
Sps	0.29^2^–0.46^3^	0.68^3^–0.80^3^	×		
Kps	0.17^n^–0.37^3^	0.20–0.40^3^	0.27^1^–0.57^3^	×	
Kw	0.16^n^–0.48^3^	0.28^2^–0.45^3^	0.30^2^–0.58^3^	0.84^3^–0.94^3^	×
Tkw	0.04^n^–0.42^3^	0.14^n^–0.31^2^	0.14^n^–0.35^3^	0.17^n^–0.26^1^	0.48^3^–0.72^3^

*Ph* plant height (cm), *Sl* spike length (cm), *Sps* number of spikelets per spike, *Kps* number of kernels per spike, *Kw* kernel weight per spike (g), *Tkw* thousand kernel weight (g)

Significant at: ^1^ *p* < 0.01; ^2^ *p* < 0.001; ^3^ *p* < 0.0001; ^n^not significant

The K-W test in the RIL-S population revealed intervals from five chromosomes to be engaged in the genetic determination of Ph (Table 3). Markers located on 2R, 4RS and 7RL were significantly associated with Ph in all vegetation seasons, thus indicating the presence of genes in these regions acting consistently in different years. Additional markers associated with the length of straw were identified on chromosomes 3RS, 3RL and 6R. As could be expected, for all of these QTLs found within the RIL-S population, the average Ph of lines representative for a maternal allele of 541 was significantly higher than that of lines carrying an allele from the paternal 2020LM (Table 3).

**Table 3:** Genomic regions indicated in the Kruskal–Wallis (K-W) test as associated with the genetic determination of quantitative traits within the RIL-S population

Trait	Chromosome	Interval/position (cM)	2008			2009			2010			2011		
			Marker ^a^	Mean value^b^		Marker^a^	Mean value^b^		Marker^a^	Mean value^b^		Marker^a^	Mean value^b^	
				M	P		M	P		M	P		M	P
Ph	2R	0.0–77.3	**XrPt390530**	121.9	103.3	XrPt507370	99.8	80.2	**XrPt390530**	96.3	81.1	XrPt389441	112.5	98.0
		122.4–122.7	**XrPt411521**	117.8	105.5	**XrPt411521**	97.5	83.2	**XrPt411521**	93.3	81.5	**XrPt411521**	109.8	98.2
	3R	0.0–10.3				XrPt345544	100.6	86.0	**XrPt349351**	96.0	82.5	**XrPt349351**	110.4	100.5
		56.0–122.4				XrPt401143	100.6	86.7	XrPt508228	94.5	80.2	XrPt390500	111.2	101.6
	4R	0.0–24.0	XrPt398487	116.1	106.5	XrPt507218	98.6	80.3	XrPt508547	94.0	77.9	XrPt506220	109.0	98.9
	6R	18.6–19.3				XrPt400431	98.3	86.6	XrPt506337	94.8	83.8			
		53.2–72.6				XrPt507720	100.3	86.4	XrPt398479	95.9	82.7			
	7R	80.2–89.0	**Xscm150**	115.0	107.9	**Xscm150**	98.4	83.7	**Xscm150**	93.9	83.1	Xopn1_667	109.8	99.0
		144.6–174.0				XrPt506256	96.6	82.9	XrPt400016	93.8	83.6	XrPt399600	110.9	99.8
Sl	2R	0.0–71.4	XrPt505357	9.23	8.06	XrPt411261	7.61	6.36	XrPt390789	8.01	6.91	XrPt411165	8.74	7.88
	4R	100.8–108.0	**XrPt508443**	8.67	8.03	**XrPt508443**	7.20	6.21	XrPt401194	7.83	6.97	**XrPt508443**	8.36	7.73
		120.9–136.1	**XrPt508623**	9.03	8.10	**XrPt508623**	7.40	6.27	XrPt399893	8.09	6.87	**XrPt508623**	8.58	7.83
	5R	135.9–148.8	**XrPt507255**	9.02	7.88	XrPt120054	7.31	6.52	**XrPt507255**	7.77	6.84	**XrPt507255**	8.60	7.58
		160.6–160.9	**XrPt390510**	9.06	8.19				**XrPt390510**	7.83	7.07	XrPt402287	8.67	7.88
Sps	2R	52.9				**XrPt507370**	25.5	23.2	**XrPt507370**	26.2	23.0	**XrPt507370**	28.5	26.9
	3R	94.5	**XrPt400848**	30.3	32.7							**XrPt400848**	27.2	29.0
	4R	6.0–24.0				**XrPt507218**	25.6	23.1	**XrPt507218**	26.3	23.0			
		100.8–155.6	XrPt400525	32.8	30.5	XrPt508623	26.0	23.0	XrPt399893	27.0	23.5	XrPt401071	29.4	27.0

*Ph* plant height (cm), *Sl* spike length (cm), *Sps* number of spikelets per spike

^a^Marker identified by the K-W test as the most effectively linked with the QTL. Markers in **bold** are those which were the most effective in at least two years of study

^b^Mean value of RILs carrying: *M* maternal allele of the marker; *P* paternal allele of the marker

In the RIL-M population, application of the K-W test for the identification of regions important for Ph performance showed equally numerous QTLs as in the RIL-S population. These QTLs were distributed on all seven rye chromosomes (Table 4). Noteworthy, regions of 2R, 3R and 7R involved in the determination of the trait were consistent with the results of the analysis of the RIL-S population, but the identified intervals were clearly shorter. Additionally, an important QTL region controlling plant height was detected on 1R, together with three less considerable loci on 4RL, 5RS and 6RL. However, a noticeable difference between both populations is that QTLs responsible for a longer straw originated from one parent (the primitive maternal line 541) in the RIL-S population, while they were derived from both parental lines in the RIL-M population.

**Table 4:** Genomic regions indicated in the Kruskal–Wallis (K-W) test as associated with the genetic determination of quantitative traits within the RIL-M population

Trait	Chromosome	Interval/position (cM)	2008			2009			2010		
			Marker^a^	Mean value^b^		Marker^a^	Mean value^b^		Marker^a^	Mean value^b^	
				M	P		M	P		M	P
Ph	1R	41.6–63.5	**XrPt401799**	102.1	93.8	**XrPt401799**	97.8	92.0	**XrPt401799**	103.6	91.7
	2R	34.3–34.5	**Xrpt509630**	102.0	93.7	**Xrpt509630**	97.4	92.4	Xrpt410940	99.4	98.3
		45.1–62.8	Xpr931L1070	103.1	94.4	Xrpt505671	98.4	92.8	Xrpt411177	100.0	97.8
	3R	56.3–60.8				XrPt507374	91.5	98.6	XrPt390681	92.7	103.8
	4R	171.4	XrPt116809	96.2	102.0	**XrPt507200**	91.8	98.5	**XrPt507200**	95.0	102.4
	5R	16.6	**XrPt401454**	102.4	96.8	**XrPt401454**	99.1	93.1	**XrPt401454**	100.1	98.3
	6R	80.8–81.8	**XrPt402229**	104.0	96.5	XrPt401893	97.9	93.8	**XrPt402229**	101.1	98.0
		145.1–172.8	**XrPt390768**	96.5	101.2	**XrPt390768**	92.2	98.6	XrPt401239	92.3	102.3
	7R	221.3–222.4	**XrPt410763**	102.1	94.3	**XrPt410763**	98.4	91.5			
Sl	2R	46.8–53.8	Xrpt505671	8.70	8.05				Xrpt390536	7.95	7.42
	3R	76.2				**XrPt507462**	6.69	7.07	**XrPt507462**	7.41	7.95
	4R	8.9–10.9	XrPt401921	8.63	8.11	**XrPt389941**	7.12	6.67	**XrPt389941**	8.03	7.32
		31.0–37.2	**XrPt402061**	8.71	8.15	XrPt507008	7.16	6.75	**XrPt402061**	8.22	7.29
		110.2	XrPt401350	8.68	8.19	**XrPt399797**	7.14	6.74	**XrPt399797**	8.09	7.43
		127.9–142.0	**XrPt400014**	8.69	8.14	**XrPt400014**	7.15	6.76	**XrPt400014**	8.14	7.37
	5R	16.4–51.7	**XrPt411244**	8.73	7.94	**XrPt411244**	7.14	6.64	XrPt398706	8.02	7.36
	6R	0.0–17.6	XrPt401102	7.98	8.77	XrPt507629	6.62	7.20	XrPt505541	7.45	7.96
		53.1–74.8	XrPt508161	8.04	8.77	XrPt390442	6.68	7.18			
		128.1–137.8				XrPt411128	6.53	7.24	XrPt506868	7.29	7.97
		153.9–166.1				XrPt400608	6.63	7.16	XrPt390768	7.27	8.06
		181.8–182.9				XrPt507299	6.51	7.26	XrPt399985	7.34	8.02
Sps	3R	111.9–122.1	XrPt505214	31.4	29.2	XrPt505592	25.3	23.8	XrPt506504	27.4	25.8
	5R	16.4–16.6	XrPt347454	31.4	29.8	XrPt401454	25.4	24.2			
		49.5–92.6	**XrPt411244**	31.4	28.9	**XrPt411244**	25.3	23.7	XrPt398706	27.5	25.6
Kps	2R	76.5	**Xrpt389304**	26.3	30.4	**Xrpt389304**	25.6	29.2	**Xrpt389304**	16.1	20.4
	3R	69.2–73.4	**XrPt344420**	26.8	29.6	XrPt507897	25.6	29.3	**XrPt344420**	15.3	20.9
	4R	166.4–188.1	XrPt116809	25.3	30.9	**XrPt508372**	24.9	29.0	**XrPt508372**	15.7	19.8
		201.6	XrPt509637	24.1	31.1	**XrPt402158**	25.3	29.3	**XrPt402158**	15.9	21.0
Kw	2R	68.7–77.8	Xrpt402364	0.72	0.85	Xrpt347771	0.60	0.73	Xrpt401519	0.32	0.43
	3R	70.6–71.6	**XrPt344420**	0.74	0.83	XrPt507897	0.62	0.72	**XrPt344420**	0.31	0.44
	4R	165.2–180.1	XrPt116809	0.69	0.87	XrPt401412	0.58	0.71	XrPt508372	0.32	0.42
		198.4–216.6	XrPt390758	0.64	0.87	**XrPt402158**	0.58	0.75	**XrPt402158**	0.31	0.44
	6R	96.5	**XrPt400777**	0.72	0.83	XrPt398472	0.60	0.72	**XrPt400777**	0.31	0.43
		120.1–128.1	XrPt399399	0.73	0.84	**XrPt401083**	0.58	0.73	**XrPt401083**	0.31	0.43
		182.9	**XrPt399985**	0.71	0.85	**XrPt399985**	0.59	0.75	**XrPt399985**	0.31	0.41
Tkw	1R	106.3–116.4	XrPt508501	25.5	29.4	XrPt506153	23.0	24.7	XrPt509026	18.5	21.4
	2R	68.7–77.8				**Xrpt402364**	22.5	25.1	**Xrpt402364**	18.2	21.5
		187.6				**Xrpt506723**	24.9	22.3	**Xrpt506723**	20.8	19.0
	4R	199.2–202.0	XrPt506651	25.8	28.0	**XrPt398716**	21.9	25.2	**XrPt398716**	18.4	21.0
	6R	81.6–120.1	XrPt509242	25.0	28.6	XrPt402208	22.1	24.9	XrPt505345	18.0	21.0
		127.0–128.1	XrPt399399	25.5	28.6	XrPt411128	22.1	25.3	XrPt509330	18.9	20.8
		182.9	**XrPt399985**	25.7	28.7	**XrPt399985**	22.3	25.4	**XrPt399985**	19.3	20.5

*Ph* plant height (cm), *Sl* spike length (cm), *Sps* number of spikelets per spike, *Kps* number of kernels per spike, *Kw* kernel weight per spike (g), *Tkw* thousand kernel weight (g)

^a^Marker identified by the K-W test as the most effectively linked with the QTL. Markers in **bold** are those which were the most effective in at least two years of study

^b^Mean value of RILs carrying: *M* maternal allele of the marker; *P* paternal allele of the marker

Sl and Sps were highly correlated in both mapping populations (Table 2). The K-W test indicated that markers associated with Sl in the RIL-S population were located on 2R, 4R and 5R. Sps was controlled by QTLs identified in the same regions of 2R and 4RL, and by additional loci on 3RL and 4RS, where QTLs for Ph were also found (Table 3).

Within the RIL-M population, markers significantly associated with Sl were indicated by the K-W test on 2R, 3R, 4R, 5R and 6R. Several intervals with markers linked to these QTLs were distributed mainly on 4R and 6R, while on the remaining chromosomes, single intervals were identified (Table 4). The K-W test revealed that Sps remained under the control of genes from one interval on 3RL and two intervals on 5R (Table 4). The number and weight of kernels per spike were controlled by QTLs located on 2R and 3R, and by two QTL regions on 4R (Table 4). Among them, a QTL located on 2R and another one on 4R were significantly associated with Tkw. Additional QTL regions responsible for Tkw were located on 1R and within three intervals of 6R. The latter three QTLs from 6R were indicated by the K-W test as being important for Kw as well.

**Fig. 1:**
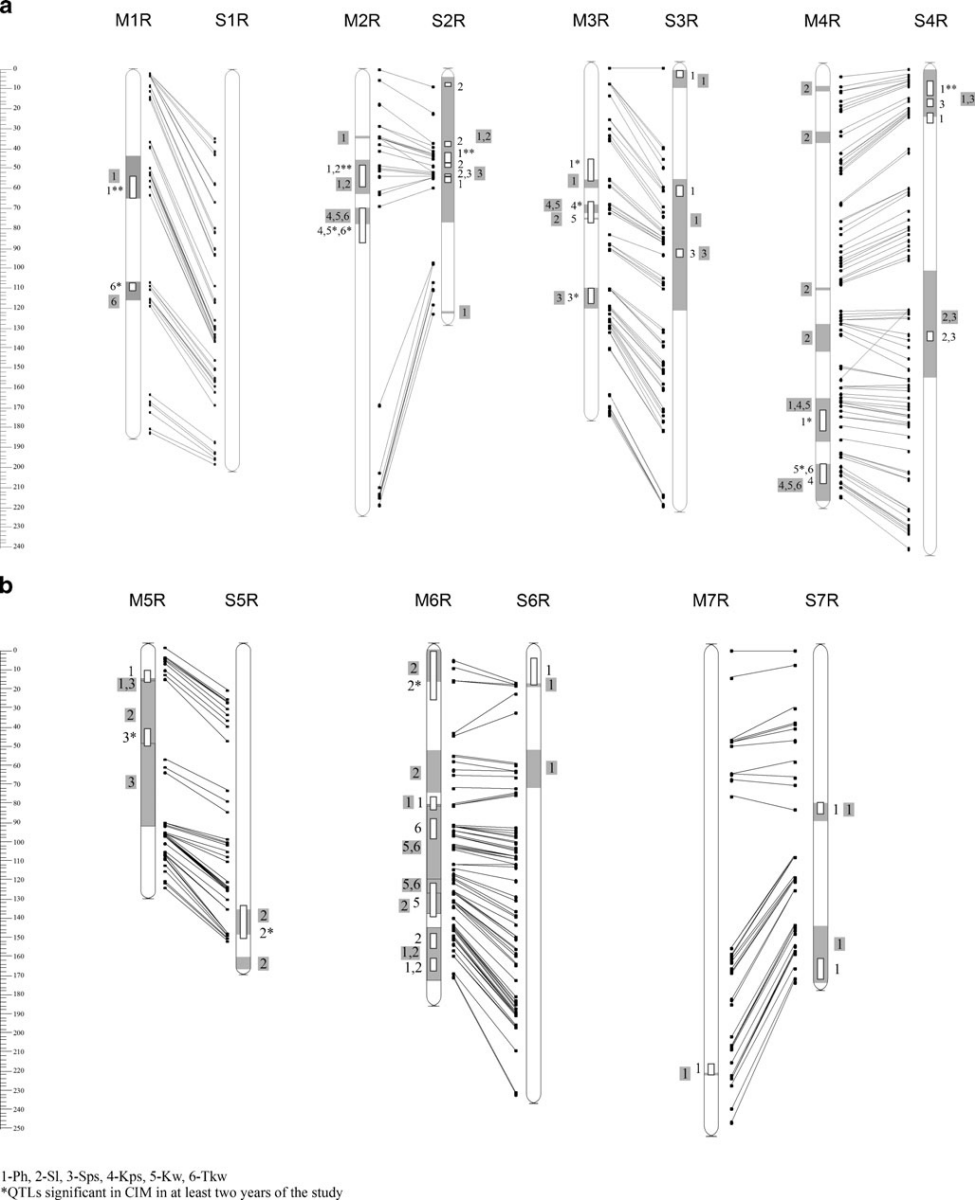
Linkage maps of rye chromosomes with the localisation of quantitative trait loci (QTLs) determining morphological traits identified with the Kruskal–Wallis (K-W) test (*grey rectangles*) and their relationships with QTLs detected with composite interval mapping (CIM) (*white rectangles*)

The CIM procedure revealed 35 QTL regions engaged in the determination of the studied morphological traits in the RIL-S population; 52 intervals carrying such QTLs were identified in the RIL-M population (all data from the CIM analysis are accessible as Electronic Supplementary Material ESM 1 and ESM 2). These regions were distributed over all rye chromosomes and most were detectable in only single years of the study. QTLs revealing major phenotypic effects with R^2^ > 10 % are listed in Table 5. The numerous group of QTLs detected via CIM remains in an agreement with genomic regions found when the K-W test was applied. Frequently, the intervals indicated by CIM were narrower. Sometimes, it allowed for the identification of more than one QTL within a given genomic region; for example, on 2R of the RIL-S population (Fig. 1). On the other hand, there were some cases where an interval revealed by CIM was wider than that found with the K-W test (Fig. 1).

**Table 5:** Quantitative trait loci (QTLs) detected by composite interval mapping (CIM) for plant height (*Ph*), length of spike (*Sl*), number of spikelets (*Sps*) and kernels per spike (*Kps*), kernel weight per spike (*Kw*) and thousand kernel weight (*Tkw*) revealing major phenotypic effect (R^2^ > 10) in populations RIL-S and/or RIL-M. QTLs in **bold** are those which were identified in the regions indicated by both CIM and the K-W test

Chromosome	RIL-S						RIL-M					
	Trait/year	Interval	Marker	LOD	a	R^2^	Trait/year	Interval	Marker	LOD	a	R^2^
1R	Sl10	39–44	XrPt506299	2.81	−0.54	10.95	Kw09	7–12	XrPt119594	3.58	−0.08	11.5
	Sl08	147–149	Xscsz732L530	2.84	0.50	10.92	**Ph09**	**52–58**	**XrPt509169**	**3.16**	**3.79**	**10.61**
	Sps08	168–173	XrPt410988	3.58	−1.77	15.61	**Ph10**	**53–63**	**XrPt399269**	**6.36**	**5.87**	**19.21**
							**Ph08**	**54–58**	**XrPt509169**	**4.73**	**5.71**	**15.93**
							Sl08	108–110	XrPt508375	3.63	−0.39	10.32
							**Tkw08**	**108–110**	**XrPt508375**	**7.19**	**−2.50**	**20.21**
2R	Sl10	36–39	XrPt390292	3.56	0.92	15.38	Sps08	22–24	Xrpt400282	3.56	−1.17	10.06
	**Ph09**	**42–46**	**XrPt509630**	**3.51**	**10.32**	**14.36**	**Ph08**	**45–57**	**Xrpt399313**	**3.54**	**4.34**	**10.61**
	**Ph11**	**45–47**	**XrPt506926**	**3.94**	**7.82**	**14.68**	**Sl08**	**52–55**	**Xrpt400417**	**5.26**	**0.49**	**14.61**
	**Ph08**	**45–46**	**XrPt401256**	**8.69**	**8.01**	**27.92**	**Tkw09**	**67–85**	**Xrpt347771**	**3.85**	**−2.01**	**12.63**
	**Sl09**	**47–49**	**XrPt509669**	**2.57**	**0.49**	**12.55**	**Tkw10**	**68–76**	**Xrpt347771**	**4.27**	**−2.23**	**14.65**
	**Sps08**	**53–54**	**XrPt400417**	**3.11**	**3.08**	**15.64**	**Kw09**	**68–81**	**Xrpt347771**	**3.10**	**−0.09**	**11.12**
	**Ph11**	**54–57**	**XrPt389556**	**5.93**	**10.56**	**25.01**	**Kw08**	**75–78**	**Xrpt389385**	**3.15**	**−0.13**	**10.26**
	Sps08	58–66	XrPt411019	2.77	1.83	13.71						
3R	**Ph10**	**2–5**	**XrPt349249**	**2.58**	**7.61**	**10.93**	Sl10	46–56	XrPt411440	2.72	0.43	10.56
	**Ph09**	**60–65**	**XrPt507739**	**4.11**	**11.54**	**19.47**	**Ph09**	**52–57**	**XrPt507374**	**5.09**	**−4.19**	**14.50**
	Sl08	92–96	XrPt400848	2.65	−0.85	13.85	**Ph10**	**54–57**	**XrPt507374**	**5.36**	**−5.00**	**15.56**
	**Sps08**	**92–96**	**XrPt400848**	**4.26**	**−2.88**	**23.53**	**Sps09**	**111–118**	**XrPt505214**	**3.60**	**0.81**	**10.67**
	Ph09	176–178	XrPt505542	2.88	14.86	11.78	**Sps08**	**112–116**	**XrPt505214**	**4.08**	**1.47**	**12.64**
4R	**Ph11**	**6–12**	**XrPt509509**	**4.25**	**7.12**	**19.90**	Kw10	55–59	XrPt509687	3.72	0.11	14.56
	**Ph09**	**7–14**	**XrPt509509**	**2.47**	**8.99**	**12.97**	Kps08	87–91	XrPt508114	3.05	6.77	10.80
	**Ph10**	**8–12**	**XrPt509509**	**4.90**	**9.48**	**25.86**	**Kw08**	**198–208**	**XrPt390758**	**4.49**	**−0.13**	**13.07**
	**Sps10**	**15–19**	**XrPt507218**	**3.49**	**1.97**	**13.8**	**Kw09**	**199–202**	**XrPt400996**	**4.61**	**−0.12**	**13.30**
	**Ph09**	**22–25**	**XrPt509687**	**2.84**	**9.07**	**10.02**	**Tkw09**	**199–202**	**XrPt400996**	**3.97**	**−2.17**	**11.24**
	Sl10	23–26	XrPt509687	2.65	0.72	11.00	**Kps08**	**206–208**	**XrPt401039**	**4.47**	**−3.94**	**12.54**
	Ph10	69–74	XrPt508114	2.86	8.97	10.8						
	Ph09	83–86	XrPt389771	3.33	18.11	17.36						
	Ph08	119–121	XrPt505925	2.7	−8.13	11.15						
	**Sl10**	**132–136**	**XrPt399893**	**3.1**	**0.50**	**11.70**						
	**Sps10**	**133–136**	**XrPt399893**	**3.46**	**2.04**	**12.88**						
	Sps08	190–193	XrPt505295	3.36	−1.93	12.14						
5R	Sl08	89–90	XrPt401037	4.39	1.40	17.68	Kps08	21–24	XrPt401095	4.43	−9.12	14.90
	Sps08	90–101	XrPt505835	2.47	1.81	11.86	Kw08	21–26	XrPt401095	3.13	−0.25	11.14
	Sps08	134–136	XrPt507255	2.34	2.26	10.73	Sl08	52–64	XrPt505276	3.52	0.43	11.52
	**Sl11**	**136–151**	**XrPt505795**	**2.37**	**0.44**	**13.26**	Sl09	67–77	XrPt507127	2.30	−0.39	10.94
	**Sl10**	**139–147**	**XrPt120054**	**2.65**	**0.54**	**11.80**						
6R	**Ph10**	**5–19**	**XrPt401305**	**2.64**	**5.71**	**11.42**	**Sl08**	**1–16**	**XrPt410992**	**5.74**	**−0.43**	**14.51**
	Sps11	49–57	XrPt400126	3.4	1.7	15.6	**Kw09**	**122–140**	**XrPt400157**	**3.73**	**−0.08**	**10.34**
	Ph08	142–147	XrPt411401	2.79	5.31	11.64	**Sl08**	**154–155**	**XrPt347758**	**5.28**	**0.74**	**17.84**
	Sl08	154–157	XrPt507753	3.06	0.82	13.15	**Sl08**	**166–167**	**XrPt390768**	**4.04**	**−0.51**	**10.69**
	Sps08	225–228	XrPt509721	2.83	1.68	12.49						
7R	Sps11	56–59	XrPt505219	3.48	−1.58	16.15	Sl09	13–30	XrPt411166	2.88	0.37	16.26
	Sl10	57–59	XrPt505219	4.35	−0.87	19.16	Ph10	53–61	XrPt400783	4.14	4.41	12.04
	Sps10	57–59	XrPt505219	3.51	−2.21	15.07						
	Sl09	65–72	XrPt401795	2.39	−0.63	10.87						
	Sps09	65–72	XrPt401795	2.51	−1.79	12.84						
	**Ph10**	**80–86**	**XrPt506902**	**2.39**	**6.14**	**10.19**						
	**Ph09**	**161–169**	**XrPt400016**	**4.03**	**8.7**	**18.4**						
	Sl11	167–171	XrPt400016	2.56	0.53	12.45						
	Sl09	170–172	XrPt344822	2.81	0.68	13.06						
	Sps09	170–172	XrPt390593	2.51	1.76	11.91						

*a* additive effect of the maternal allele

The parental lines of the RIL-S population represented a lower genetic similarity than the parents of the RIL-M population, but the number of intervals containing QTLs controlling traits analysed simultaneously in both mapping populations (Ph, Sl, Sps) seems to be comparable or even more numerous within the RIL-M population (Fig. 1). Significant differences between both populations become more visible when the phenotypic effect of QTLs is considered. This effect is indicated in the CIM method by calculating the determination coefficient (R^2^). The majority of QTLs detected in the RIL-S population revealed a strong impact on plant phenotypes. Out of 54 QTLs (ESM 2), 48 had R^2^ > 10 % (Table 5). With regard to the RIL-S population, values of the additive effect were usually positive, indicating that alleles increasing the analysed traits originated from the maternal inbred line 541. On the map of the RIL-M population, only 62 of 96 QTLs were classified as important (Table 5). Among them, QTLs controlling plant height were detected in all years of the study on 1R and some additional loci for this trait were located on 2R, 3R (confirmed in at least two vegetation seasons) and 7R. The phenotypic effect of the QTL for Ph detected in 2 years on the long arm of 4R (Fig. 1) was considered a minor one.

## Discussion

The proper estimation of phenotypic values is very important for QTL mapping. However, field experiments were performed within 3–4 years, but the phenotypes of individual plants grown on non-replicated plots were analysed in one location only; therefore, the effect on the results of random factors cannot be excluded. Additionally, Melchinger et al. (1998) showed that studies of mapping populations composed of less than 200 genotypes do not allow for the detection of all QTLs, especially for quantitative traits with low heritability. The statistical analysis shown in Table 1 proved a significant role of the genetic component of variance. In order to minimise the influence of random factors, only QTLs constantly detected in two or more years of our study were considered reliable. A significant group of QTLs was detected with the use of both applied statistical methods. Application of the non-parametric K-W test for the detection of QTLs was inspired in this study by the needs of breeding practice. This rank sum test involves studying a number of single genetic markers one at a time (independently from other markers on the genetic map). Thus, it is less precise in the detection of the localisation of a given QTL, but allows the easy identification of the most effective markers for MAS and gives direct information about the average phenotypic effect that is capable of being obtained during selection. Genomic intervals indicated to be important by the K-W test for the studied traits were relatively long, but mostly consistent in subsequent years of the study.

In the CIM procedure, the QTL effects are explained by a normal mixture model. At a given location in the genome, the presence of QTLs is estimated by the LOD score, i.e. the likelihood ratio of the mixed model compared to a single normal distribution. This technique can sometimes produce spurious LOD score peaks in genome regions with low genotypic information. These peaks are not indicative of a QTL and, very often, are an effect of a better fit of a mixture of normal distributions than a single normal distribution. An advantage of the LOD method appears to be the possibility of scanning genomic regions (intervals) located between genetic markers (Lander and Botstein 1989), which increases the precision of QTL mapping. In general, for normally distributed traits, the results of QTL mapping with the use of a non-parametric test of single markers (K-W test) and interval mapping based on the LOD method should be consistent with a remarkably higher statistical power, in favour of the CIM method. If these results between the two approaches differ significantly, they should be interpreted with considerable caution (Kruglyak and Lander 1995) and may suggest that the distribution of the residuals deviates from normality assumptions. The probability of this situation is greater if the phenotypic evaluation was based on experimental designs performed in a completely random model, without blocks, and in such circumstances, a remarkable part of interplant variation caused by non-genetic factors (e.g. soil heterogeneity) is not extracted from the residuals. On the other hand, recently published research showed that all currently achievable software applying LOD scores for QTL mapping can generate “false-positive” QTLs, even if the data come from computational simulation (Su et al. 2013). The application of two statistically independent methods (and different software) in our study allowed for the indication of highly reliable QTLs controlling plant morphology in rye. There are, however, also numerous loci detectable by only one of the applied methods and their localisation needs to be verified in future studies.

Phenotypic variation in all of the traits studied in this research was significantly affected by an interaction between years (environments) and genotypes. Reasonable utilisation of molecular markers in MAS needs the exploration of stable QTLs which are detected in different environments, in different genetic backgrounds and those revealing pleiotropic effects on more than one trait (Wang et al. 2009). The CIM procedure mainly revealed the QTLs detectable in only one vegetation season, but *loci* revealing more stable phenotypic effects were also identified. Some of these showed a pleiotropic effect, but were usually not congruent in both mapping populations. Even if two mapping populations were found to have a common parental line, congruently detected QTLs are observed relatively seldom in rye (Miedaner et al. 2012) and wheat (Cui et al. 2011, 2012).

The first studies on the localisation of QTLs determining morphological traits (Börner et al. 2000; Milczarski and Masojć 2003) were performed with the use of two independent mapping populations, both of which carried a dwarfing gene, Ddw1. The results indicating 5RL as the most significant loci for the determination of morphological traits (Ph, Sl, Tkw, Kps) were probably due to the pleiotropic activity of Ddw1. In the present study, a set of QTLs controlling morphological and yield-related traits in both of the analysed mapping populations was revealed on all seven chromosomes of rye and a concentration of QTLs affecting the traits on the distal part of the 5RL chromosome was not observed. In the RIL-M population, QTLs were evenly distributed along the 5R chromosome. In the RIL-S population, two intervals with loci controlling the spike length and number of spikelets per spike were found on 5RL, but other regions of the genome seem to be much more abundant in QTLs that are important for plant morphology in this population. Namely, the largest concentration of highly expressive loci controlling plant height and spike morphology in the RIL-S population was observed on 2R, where some QTLs were congruently detected in the RIL-M population. This proximal region of 2R was considered by Börner et al. (2000) to be important for the determination of quantitative traits in rye. There are several genes that are important for agronomical traits on the wheat 2A (Yao et al. 2009), which may coincide with those on rye 2R, taking into account a high co-linearity between 2R and the proximal region of wheat 2A (Devos et al. 1993b). In our study, numerous QTLs on the remaining rye chromosomes were distributed more evenly in both of the mapping populations studied.

The genetic architecture of some complex agronomic traits examined in test-cross populations of rye has recently been published by Miedaner et al. (2012). The analysis of quantitative traits with the use of test-crosses is often performed for outbreeding species, because it allows an inbreeding depression to be avoided and field experiments to be designed based on highly viable genotypes. An additional advantage for the application of test-crosses is that this method is more compatible with the practice of hybrid breeding, but the results of such studies are always strongly affected by the choice of a testing genotype. Research based on the phenotypes of RILs per se better reflects the real (theoretical) value of the studied lines, but for complex traits, these observations may have a limited value for breeding applications. Therefore, interesting findings can be derived from a comparison of our study with those presented by Miedaner et al. (2012). In the study by Miedaner et al. (2012), both populations used for test-crosses were developed on the basis of plant resources adopted for breeding. Since Ph and Tkw were analysed both by Miedaner’s research group and in our study, the results concerning these traits can be compared. The genomic regions responsible for Ph in the RIL-S and RIL-M populations were detected on the 2R, 3R and 7R chromosomes. Additionally, we found within the RIL-M population intervals for Ph located on 1R, 4RL and 5R, as well two QTLs for Tkw located on proximal regions of 1RL and 6RL. Similar chromosomal locations were indicated as being significant for Ph and Tkw by Miedaner et al. (2012). On the other hand, QTLs for Ph mapped on 3RS, 4RS and 6R were recorded exclusively within the RIL-S population and a QTL for Tkw on 4RL was only recorded within the RIL-M population.

The present study contributes new data about the very complex mechanism of the determination of morphological traits in rye. The mapping of QTLs controlling Sl, Sps, Kw and Kps was performed on high-density maps of rye for the first time. Additionally, novel QTLs for Ph and Tkw, not reported by Miedaner et al. (2012), were found. Characteristics of QTLs showed that none of them had a large genotypic effect, which leads to the conclusion that genomic selection (simultaneous analyses of numerous loci and their application for the selection of genotypes) should be preferred to the pyramiding of genes by progressive marker-assisted selection in single locus for improving these traits in the breeding programmes.

In conclusion, it seems that, in contemporary commercial breeding programmes, a genetic diversity is rather limited to agronomically valuable genotypes and, considering this, the examined RIL-M population compared to the RIL-S population better reflects variation occurring in currently selected rye materials. As was proven in this study and in previous research on rye (Myśków et al. 2001, 2010), parental lines with related pedigrees can still reveal genetic variation sufficient for the construction of mapping populations, the detection of QTLs for different traits and the selection of valuable strains for commercial programmes. Markers linked with QTLs revealing a moderate phenotypic effect present in the currently exploited breeding resources may be more important for breeding practice than markers linked with apparently more efficient alleles occurring only in agronomically non-adopted genotypes.

## Electronic supplementary material

Below are the links to the electronic supplementary material.

ESM 1 (PDF 9547 kb)

ESM 2 (XLS 79 kb)
