# Supplementary material for: The application of high-density genetic maps of rye for the detection of QTLs controlling morphological traits
Source: J Appl Genet. 2013 Dec 3;55(1):15–26. doi: 10.1007/s13353-013-0186-5 (PMC3909618; doi:10.1007/s13353-013-0186-5)
Supplement: Supplementary file 1 — (PDF 9547 kb) [file 13353_2013_186_MOESM1_ESM.pdf]

The application of high-density genetic maps of rye for the detection of QTLs controlling morphological traits

ESM 1. Linkage maps of chromosomes 1R-7R and the localization of QTLs determining morphological traits within the populations of RIL-M and RIL-S detected with Composite Interval Mapping. Lines between chromosomes connect the positions of corresponding markers.

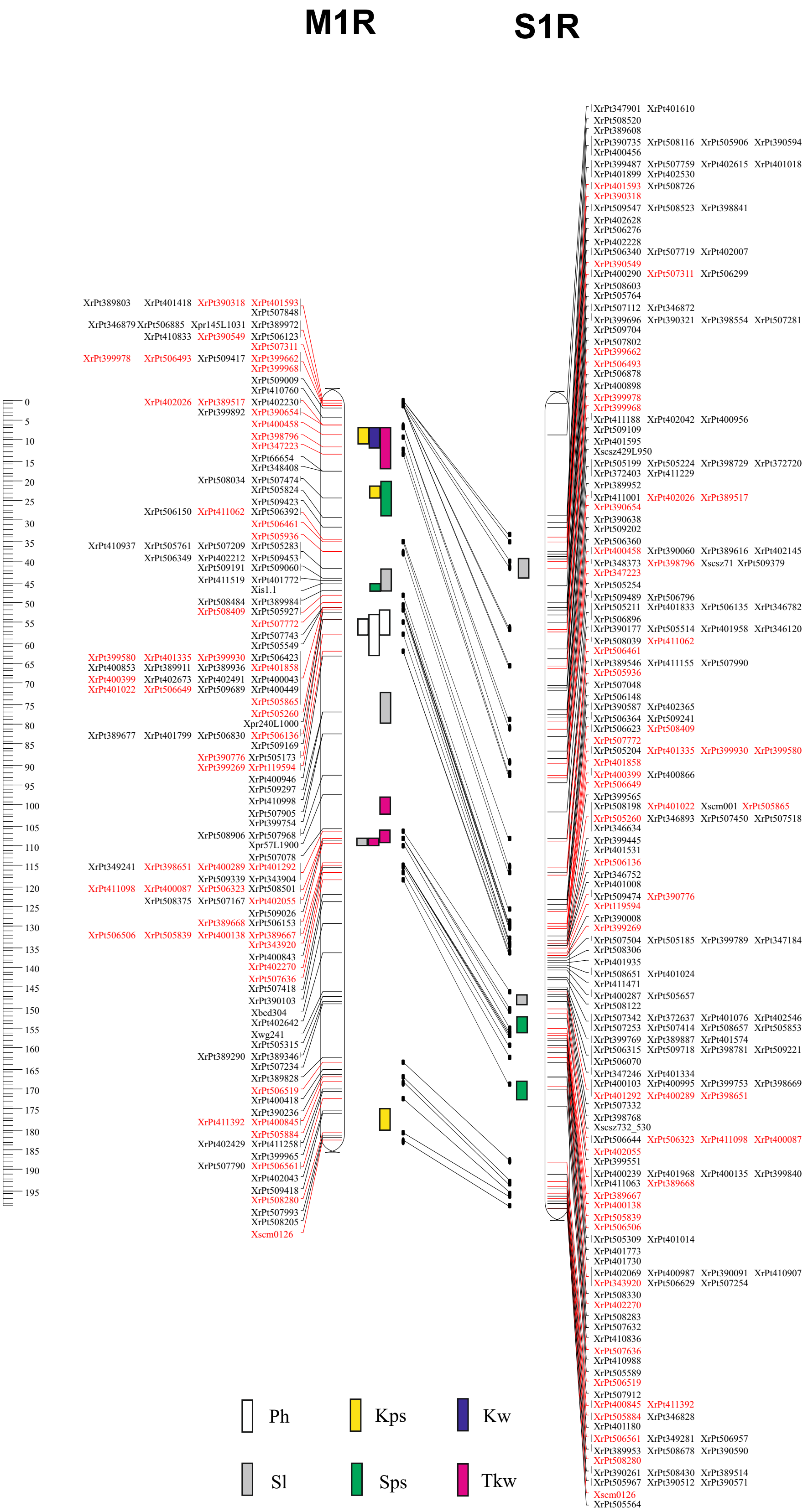

M1R
S1R
0
5
10
15
20
25
30
35
40
45
50
55
60
65
70
75
80
85
90
95
100
105
110
115
120
125
130
135
140
145
150
155
160
165
170
175
180
185
190
195
Ph
Kps
Kw
Sl
Sps
Tkw

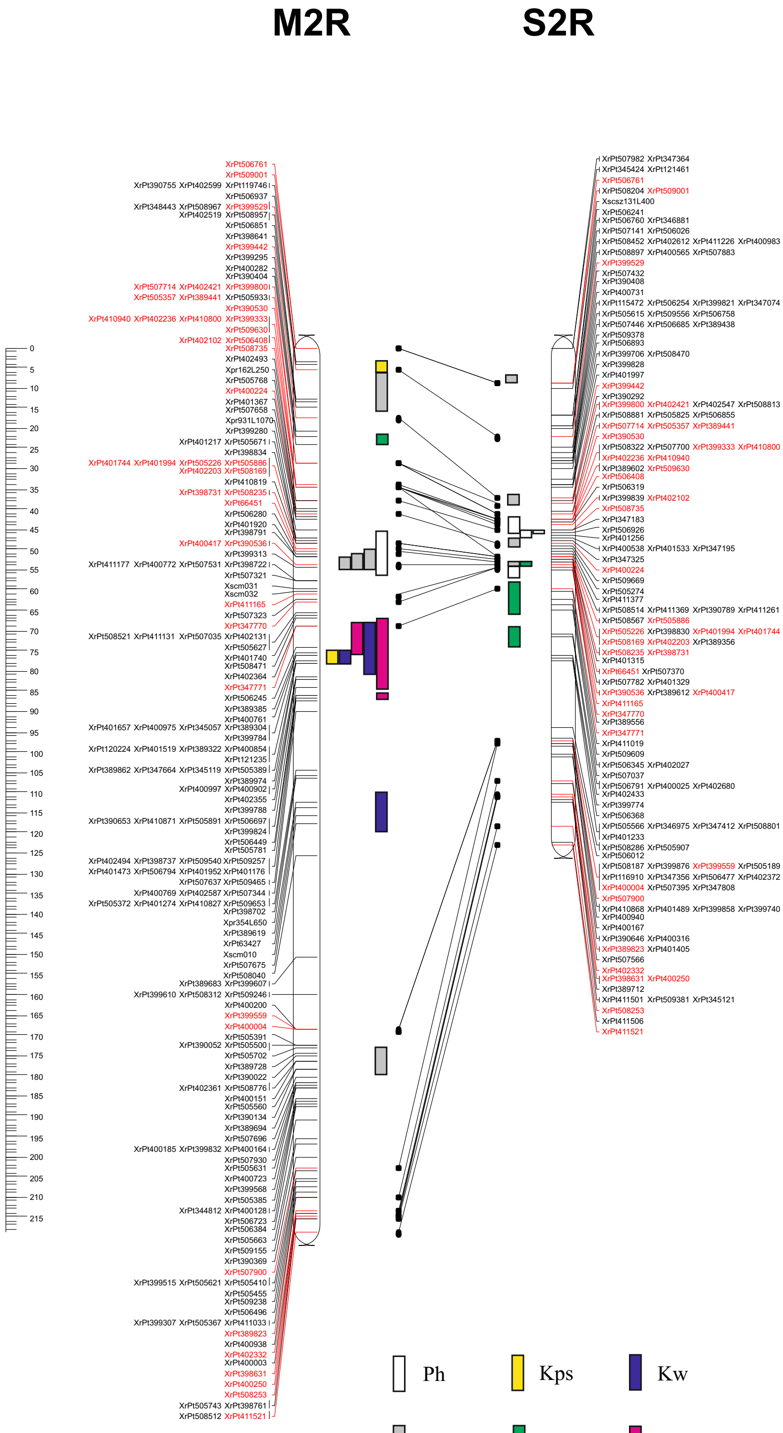
M2R
S2R
Ph
Kps
Kw
Sl
Sps
Tkw

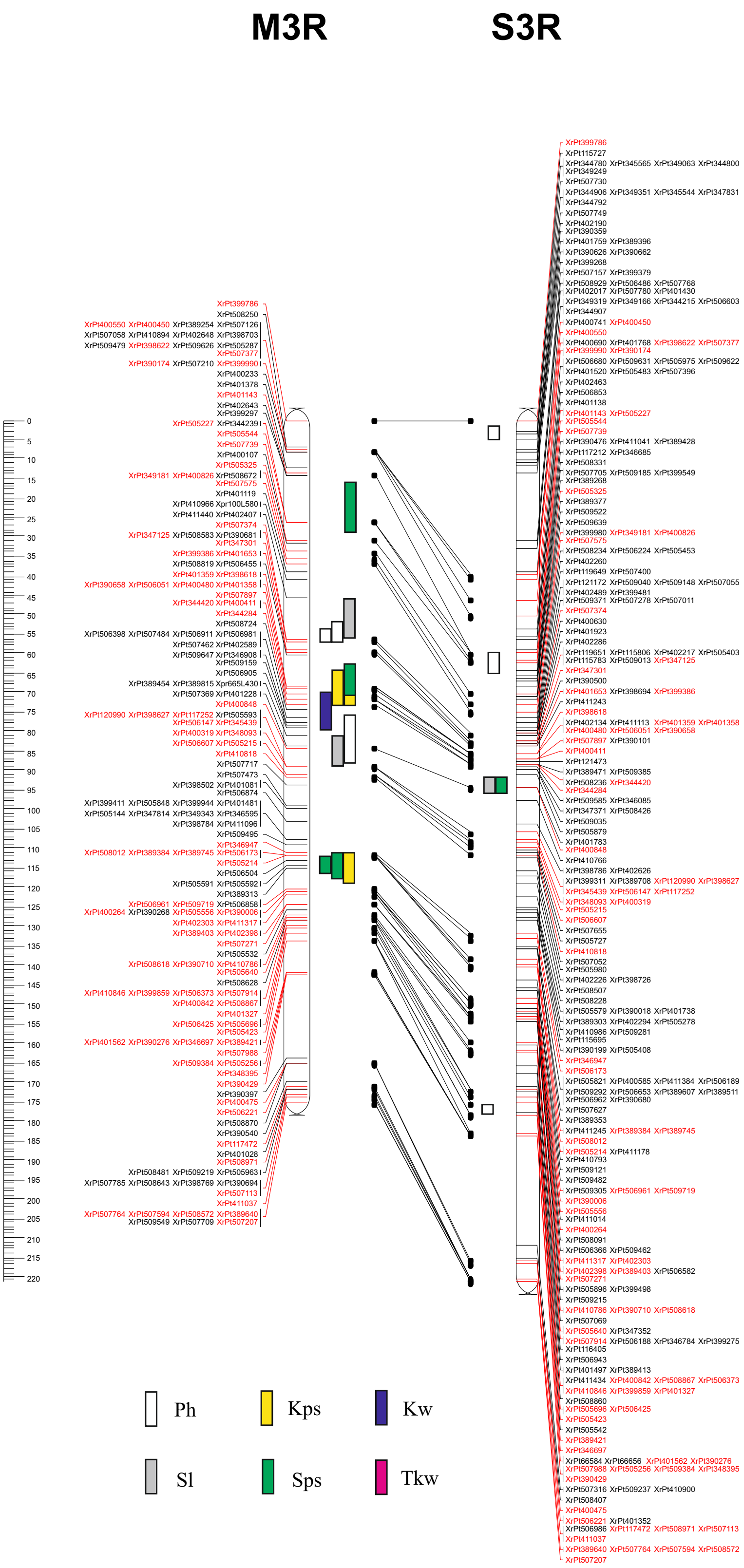
M3R
S3R
Ph
Kps
Kw
Sl
Sps
Tkw

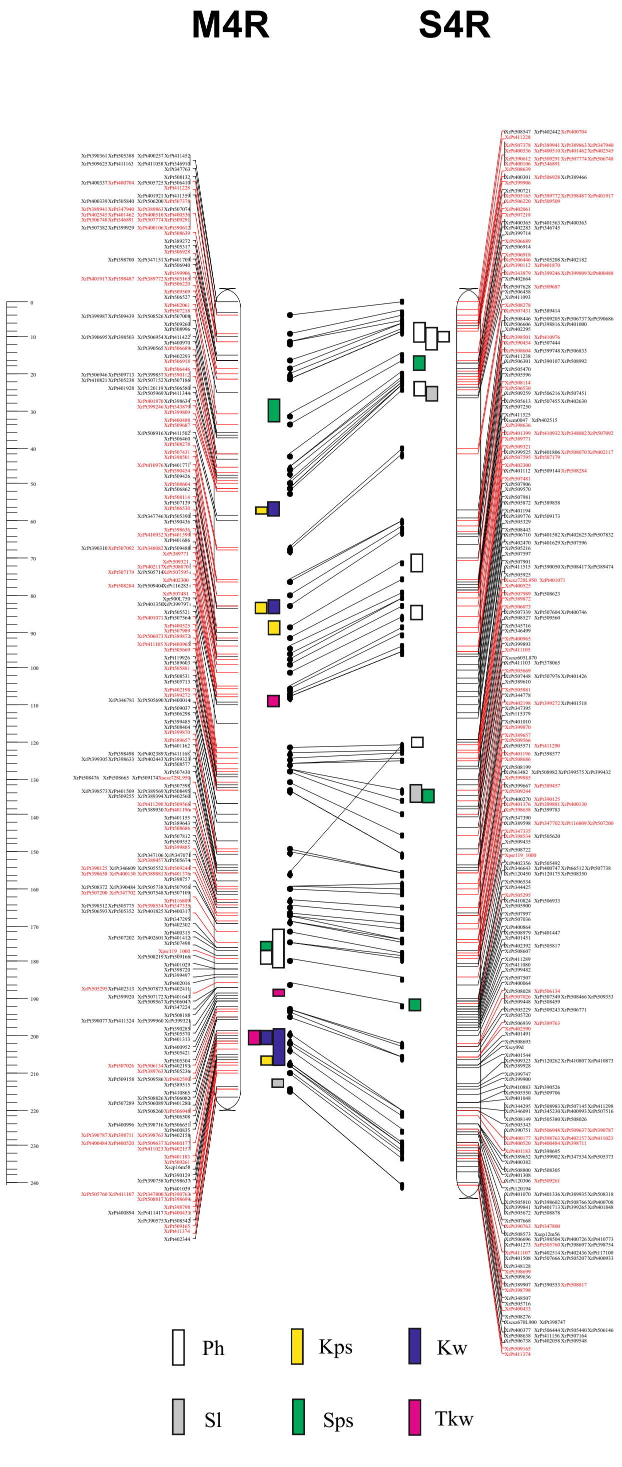
M4R
S4R
Ph
Kps
Kw
Sl
Sps
Tkw

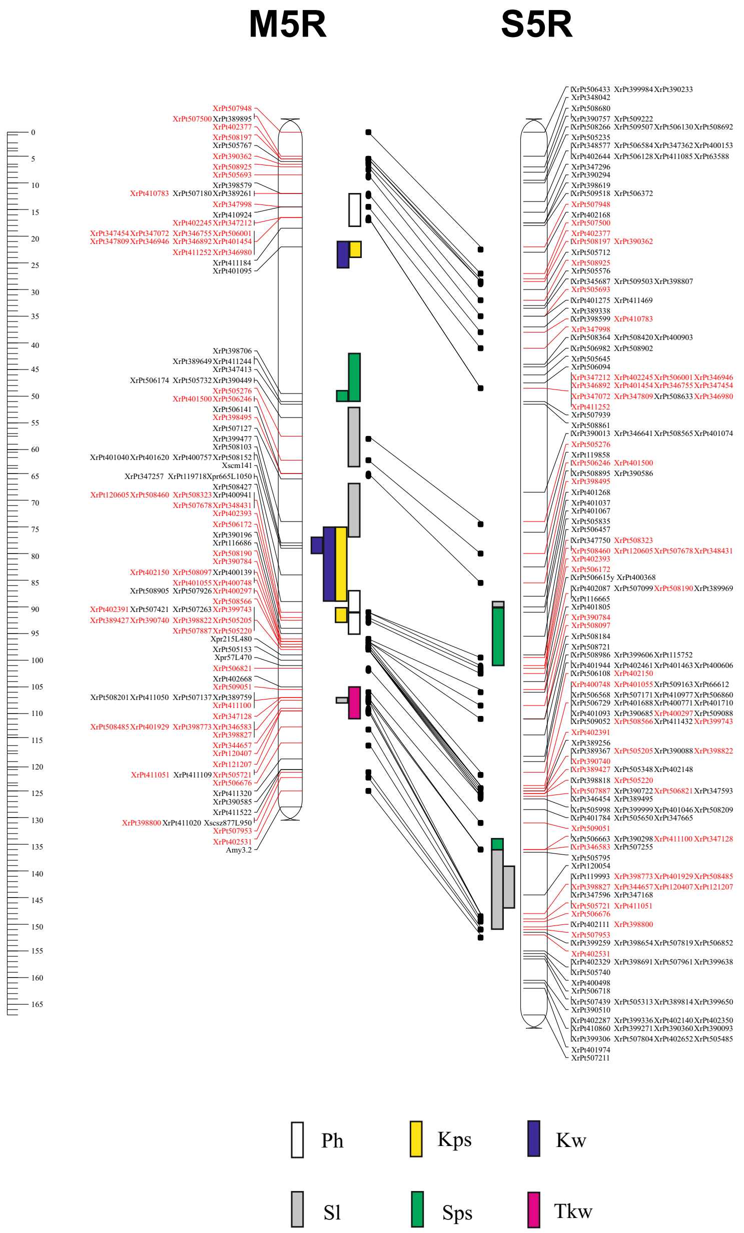

M5R
S5R
Ph
Kps
Kw
Sl
Sps
Tkw

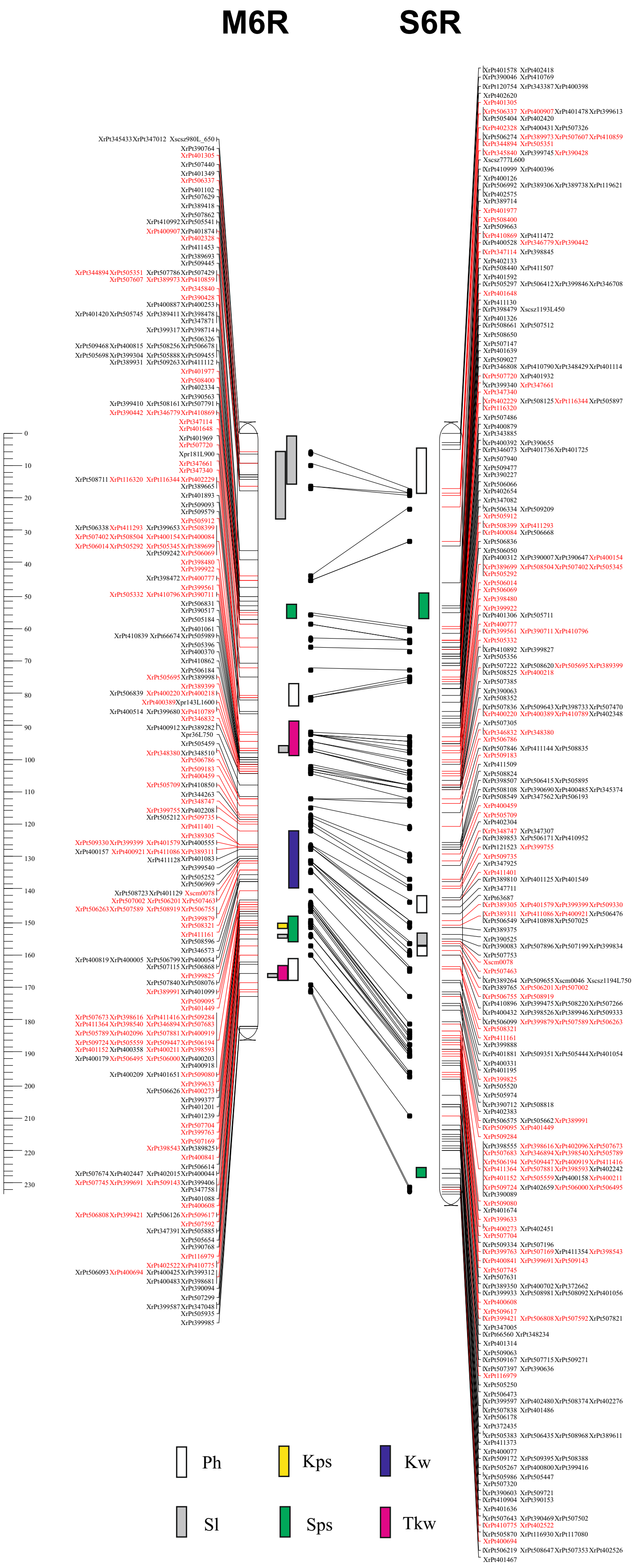
M6R
S6R
0
10
20
30
40
50
60
70
80
90
100
110
120
130
140
150
160
170
180
190
200
210
220
230
Ph
Kps
Kw
Sl
Sps
Tkw

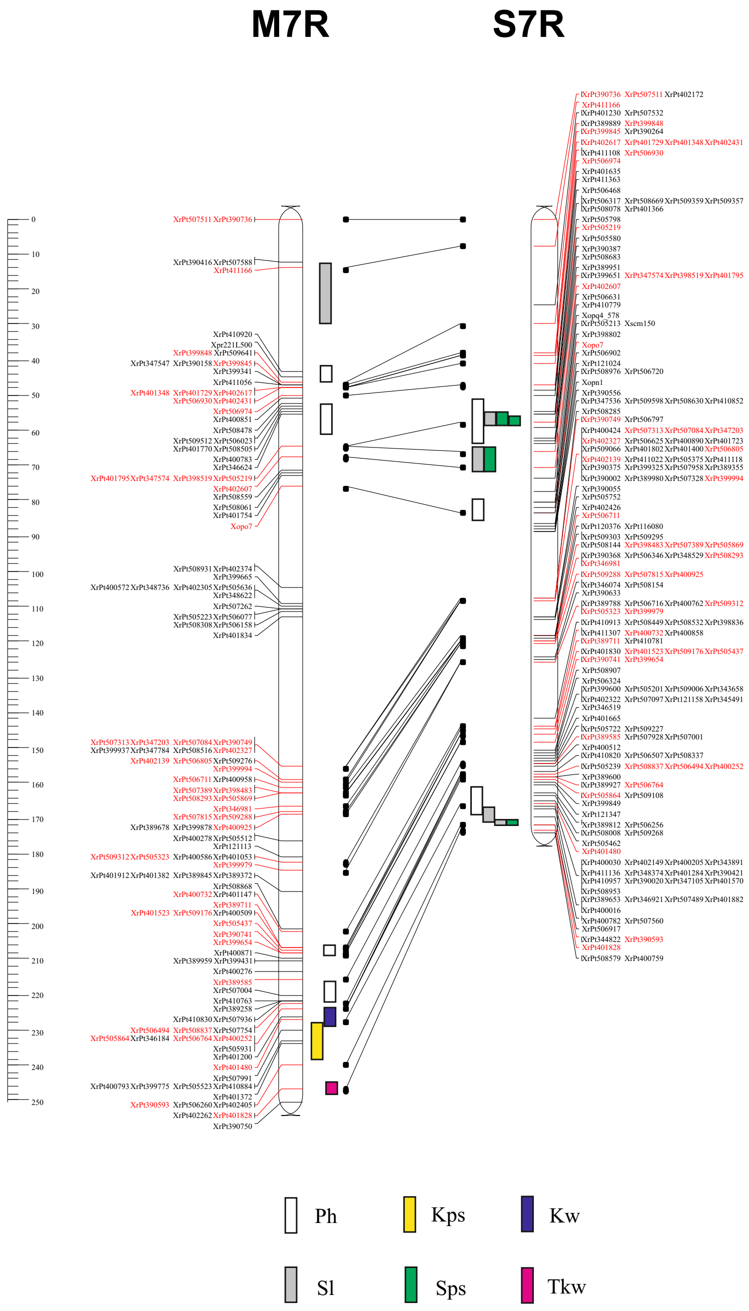
M7R
S7R
Ph
Kps
Kw
Sl
Sps
Tkw
